# Ketamine Use in Hysterosalpingography (the Jimah Procedure): A Follow-Up of Bilateral Tubal Evaluation of 27 Infertile  Women  at a Teaching Hospital, Ghana

**DOI:** 10.1155/2021/6657137

**Published:** 2021-04-17

**Authors:** Bashiru Babatunde Jimah, Anthony Baffour Appiah, Benjamin Dabo Sarkodie, Dorothea Anim

**Affiliations:** ^1^Department of Medical Imaging, School of Medical Sciences, University of Cape Coast, Cape Coast, Ghana; ^2^Ghana Field Epidemiology and Laboratory Training Programme, University of Ghana, Accra, Ghana; ^3^Department of Radiology, University of Ghana, School of Medicine Sciences, Accra, Ghana; ^4^Department of Radiology, Korle Bu Teaching Hospital, Accra, Ghana

## Abstract

**Background:**

Pain, anxiety, and distress are common in radiological investigations including hysterosalpingogram (HSG). Studies suggest that sedation allows patients to better tolerate diagnostic imaging and image-guided procedures by relieving anxiety, discomfort, and pain. This study aimed at assessing the safety and effectiveness of ketamine use in HSG and the proportion of true positive bilateral tubal blockage during HSG using the Jimah Procedure.

**Methods:**

We performed repeated HSG workup under IV ketamine (20–40 mg/mL) sedation for 27 infertile women at the Cape Coast Teaching Hospital. The exclusion criteria included unilateral tubular blockage, acute infection of the vagina or cervix, active vaginal bleeding, glaucoma, and high blood pressure at the time of the study. Data were entered with Microsoft Excel and analyzed using SPSS version 21.

**Results:**

A total of 27 patients (age range: 25–48 years) previously diagnosed of bilateral tubal blockage or spasm were enrolled for the repeat HSG procedure. The median age was 34 years (IQR: 32–37), while secondary infertility (20) (74.1%) was the commonest indication. None of the patients reported of pain or distress during or after the procedure. Two (7.4%) women vomited after HSG. Twelve patients (44.4%) had bilateral tubal blockage (true positive), while tubal patency was seen in 15 (55.6%) patients on HSG under ketamine sedation.

**Conclusion:**

This study found IV ketamine sedation produces profound anesthesia and analgesia and eliminates tubal spasm. We recommend that radiologists in developing countries should consider sedating patients during HSG and documenting observations and patients' feedback to help assess safety and effectiveness in local settings.

## 1. Introduction

Pain, anxiety, and psychological and physical distress are likened to radiological investigations including hysterosalpingogram (HSG) [1–3]. These complications can be ameliorated by the use of sedation and analgesia [1]. Although HSG is noninvasive, patients' anxiety and psychological and physical distress might interfere with the procedure and subsequent outcome [1, 2]. However, sedation in HSG procedures is rare in low-middle-income countries (LMICs) as many radiologists consider it as noninvasive [3]. For instance, sedation under HSG recorded the lowest proportion (8.5%) compared to computed tomography (CT) (65.3%), magnetic resonance imaging (MRI) (63.6%), and interventional radiology (16.1%) practices among Nigerian radiology residents [3]. Little information exists on the use of sedation in HSG procedure in Sub-Saharan Africa and Ghana. This finding suggests that radiologists are not sedating their patients during HSG or failing to document local experience to inform practice. Studies suggest that sedation allows patients to better tolerate diagnostic imaging and image-guided procedures by relieving anxiety, discomfort, and pain [1–3].

The commonest indication for HSG is infertility, usually to examine the fallopian tubes and uterine cavities [2, 4–8]. Tubal blockage is the commonest abnormality seen during HSG [2, 4, 8]. A prior study to assess the spectrum of findings on HSG in 203 women with infertility [4] revealed that forty-four (51.3%) women with abnormalities had fallopian tube blockage including bilateral blockage, right unilateral proximal blockage, and left unilateral proximal blockage. To confirm these findings, the procedure was repeated under ketamine sedation for some patients using strict inclusion and exclusion criteria to ascertain the incidence of true bilateral tubal blockage.

Ketamine hydrochloride is a dissociative anesthetic agent. It is rapidly acting and produces profound anesthesia and analgesia [9]. The half life of ketamine is 45 minutes.

It has dissociative action and partial agonism on opiate receptors, permitting the performance of painful procedures in a consistent state of sedation and patient comfort [9]. Ketamine also relieves patients' anxiety and minor psychological and physical distress, as documented in previous studies [10, 11]. Common side effects of ketamine include nausea, vomiting, dizziness, diplopia, drowsiness, dysphoria, and confusion [9].

But World Health Organisation document reveals that chronic use of ketamine is commonly associated with slurred speech, dizziness, blurred vision, palpitations, chest pain, vomiting, and insomnia [12] and, hence, use with caution.

We hypothesized that administration of IV ketamine produces anesthesia and analgesia and relieves tubal spasm. We determined the safety and effectiveness of ketamine and the proportion of true positive bilateral tubal blockage during HSG under ketamine sedation.

## 2. Subjects and Methods

### 2.1. Study Design

This is a cross-sectional study involving 27 women (aged 25–48 years), undergoing repeated HSG under ketamine sedation for bilateral tubal blockage over 2 years (January 2018 to December 2020) at the Department of Imaging, Cape Coast Teaching Hospital, Ghana. This represents 13.3% of the 203 women who presented for the HSG procedure and were initially diagnosed with bilateral tubal blockage. The initial study [4] involving 203 women underwent the traditional method of performing HSG in Ghana, which does not include ketamine sedation. The 27 patients, thus, had the new procedure called the “Jimah procedure” which is a modified version of the traditional procedure and involves IV ketamine use.

### 2.2. Inclusion Criteria

1. Diagnosis of infertility (primary or secondary)
2. HSG diagnosis of bilateral tubal blockage

### 2.3. Exclusion Criteria

1. Women with acute infection of the vagina or cervix
2. Women with active vaginal bleeding
3. High blood pressure at the time of the procedure
4. Glaucoma
5. Allergy to contrast medium or ketamine

### 2.4. The Jimah Procedure: “Modified Traditional HSG Procedure”

Prior to the procedure, informed consent was obtained from each patient. Blood pressure, pulse, respiratory rate, and oxygen saturation were documented. Known allergies and underlying chronic conditions were documented. Patients were asked to abstain from alcohol from the start of their menstrual cycle. HSG was performed in the preovulatory phase of the menstrual cycle as an outpatient procedure between the 5^th^ and 10^th^ day of the menstrual cycle. The patients were placed in lithotomy position. The vulva was cleaned with antiseptic solution. Vaginal speculum was passed to visualize the cervix. Uterine sound was used to assess the patency of the cervical canal. Intravenous ketamine was administered until the patient's pulse begins to increase, at which point the patient becomes unresponsive. The dosage of ketamine and initial and final finger pulse were recorded. *It is important to visualize the cervix and assess its patency prior to sedation because, in certain situations, the cervix may not be visualised and/or there may be severe cervical canal stenosis, making it difficult to cannulate the cervical canal.* The cervical cannula was inserted after air bubbles have been expelled. Approximately 10–20 ml of water-soluble contrast medium (iopamidol) was injected manually through the cannula under fluoroscopic guidance. Two supine hysterograms were taken. The first image was obtained during the early filling of the uterus and used to evaluate any filling defects or uterine contour abnormality. The second image was taken with the uterus and fallopian tubes fully distended and free intraperitoneal spillage of contrast material seen. An oblique hysterogram was taken in uncertain cases, and no delayed images were taken. The recovery time was between 40 and 60 minutes. Patients are advised not to drive or use heavy machinery for the next twenty-four hours.

### 2.5. Data Analysis and Interpretation of HSG

Patient's demographic information and HSG findings were entered into Microsoft Excel and later exported with Statistical Package for the Social Sciences (IBM SPSS version 21) for the analysis. Data were summarized with descriptive statistics using median (interquartile range, IQR), frequencies, and percentages. Fisher's exact test was used to assess the association between age group of women and HSG findings at a *p* value <0.05.

HSG is considered normal when there is a free flow of contrast medium into the peritoneal cavity and normal uterine outline. HSG was considered abnormal when there was evidence of either unilateral or bilateral tubal obstruction or uterine cavity abnormality [4].

### 2.6. Ethical Considerations

This study was approved by Ethical Review Committee of the Cape Coast Teaching Hospital (CCTHERC). Written informed consent was obtained after the nature of the study was adequately explained to the clients. Clients were assured of data security and confidentiality.

## 3. Results

### 3.1. Demographic and Clinical Presentation of Women

In total, 27 patients (age range: 25–48 years) were enrolled for repeated HSG workup under 20–40 mg IV ketamine sedation with a diagnosis of bilateral tubal blockage or tubal spasm. The characteristics of the 27 patients are shown in Table 1. The median age was 34 years (IQR: 32–37). Most of them aged between 25 and 34 years 14 (51.9%), had secondary infertility 20 (74.1%), and received 20 mg of IV ketamine 18 (66.7%). The median baseline pulse rate was 65 bpm (IQR: 64–67) which increased after IV ketamine sedation (98 bpm) (IQR: 95–100). None of the patients complained of pain during or after the procedure, but 2 (7.4%) patients experienced vomiting after administering IV ketamine.

### 3.2. Follow-Up HSG Findings under Ketamine Sedation

**Figure 1:**
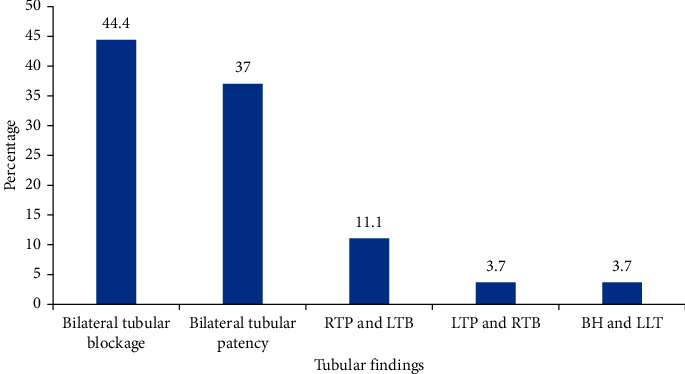
Distribution of tubal finding on HSG among 27 infertile women under ketamine sedation. RTP: right tubal patency; LTB: left tubal blockage; RTB: right tubal blockage; LTP: left tubal patency; BH: bilateral hydrosalpinx; LLT: leakage left tube.

In Figure 1, the distribution of tubal findings on HSG among 27 infertile women is summarized. Bilateral tubular blockage was seen in 12 (44.4%), while tubular patency was seen 15 (55.6%) on HSG under ketamine sedation.

The distribution of HSG findings by age group of infertile women is shown in Table 2. Bilateral tubal patency was commonly diagnosed among women aged 25–34 years (80%). Most women with bilateral tubal blockage were more than 35 years old (58.3%). All the uterine fibroids (3) (100.0) were seen among women aged 35 years and above. Only uterine findings were significantly associated with the age group of the women (*p*=0.041).

The proportion of true positive bilateral tubal blockage among the 27 women was 44.4% (*n* = 12) (Table 3).

## 4. Discussion

Hysterosalpingography (HSG) is a frequently utilized diagnostic procedure to assess tubal patency and intrauterine anatomical defects in the infertility workup. However, tubal spasm, pain, anxiety, and distress interfere with patients' cooperation and outcome of the procedure [1–3]. This study involved twenty-seven women previously diagnosed of bilateral tubal blockage or spasm on HSG. Most of the women were within the third decade (25–34 years) (51.9%). Secondary infertility was the commonest indication (74.1%). True bilateral tubal blockage was reported among twelve (44.4%) women. None of the patients reported pain or distress during the repeat procedure, but two (7.4%) of the women experienced ketamine-induced vomiting.

There is limited literature on the safety and effectiveness of ketamine use in HSG workup in Sub-Saharan Africa. This outlines the essence of documentation and reporting of local experience of performing HSG under ketamine sedation at the Cape Coast Teaching Hospital.

The WHO 36^th^ Expert Committee on Drug Dependence (ECDD) documented evidence on the pharmacodynamics of ketamine which showed that IV ketamine has the potential to cause changes in heart rate, cardiac output, and blood pressure [12]. The safety and effectiveness of ketamine to sedate prehospital and emergency department (ED) patients with undifferentiated agitation have been reported in a systematic review and meta-analysis [13]. Likewise, there have been several reports on ketamine use in clinical procedures and trials among children and adolescents in developed countries [11, 14–20].

In this study, intravenous ketamine sedation was generally safe and effective in alleviating pain and stress [3, 11, 13, 21, 22]. Severe pain was reported (pain score >8) in the earlier HSG procedure without ketamine sedation. None of the patient's complained of pain during the repeat with ketamine. No tubal spasm was diagnosed as compared to the previous procedure.

Nonetheless, like previous study [13], we found that two of our patients had ketamine-induced vomiting, which is a known common side effect. Previous study reported no adverse effect with ketamine use during biopsies [16]. Only few studies have reported some adverse effects of ketamine for moderate-to high-invasive procedures in children [11].

Without sedation, bilateral tubal blockage could be due to tubal spasm [6]. Similar to a previous study by Jimah et al. [4], bilateral tubal blockage was the commonest tubal abnormality seen in this study. However, injection of IV ketamine led to high incidence of tubal patency among the studied patients.

Compared with the previous study, HSG under ketamine sedation has moderate sensitivity but relatively high specificity. This was seen in the current study where the proportion of tubal patency was 55.6% (false positive) and bilateral tubal blockage was 44.4% (true positive). The false positive indicates that more than half of our patients were hitherto misdiagnosed as bilateral tubal blockage due to pain. Compared to a study done in Benin Republic, the proportion of tubal patency was twice higher [7]. Generally, this implies that sedating patients during HSG produces a more accurate fallopian tubal finding [6]. Potentially, failure to sedate patients for HSG can lead to misdiagnosis of tubal patency as blockage due to cornual spasm. These findings support the hypothesis that administration of IV ketamine reduces tubal spasm and enhances evaluation of tubal conditions. The challenge in implementing this new procedure in most developing countries is monitoring patients after procedure. Most radiology departments do not have dedicated recovery wards. However, critical assessment can identify unused spaces that can be converted to fit such a purpose.

Common limitation seen in this study is inadequate sample size, bilateral tubal blockage is rare, and only one of the tertiary hospitals in Ghana was involved. Therefore, the study findings might not be a true incident of bilateral tubal blockage among infertile women in Ghana. There is no local standard operation procedures (SOPs) on ketamine used in radiologic procedures. However, this study has provided preliminary data to inform radiology practice in the local setting and provided a new method for performing HSG in Ghana.

## 5. Conclusion

This study found that intravenous ketamine sedation produces profound anesthesia and analgesia and eliminates tubal spasm. Administration of ketamine helps reveal false tubal blockage observed on HSG without sedation. Patients with true tubal blockage can undergo tubal recanalization. We recommend that radiologists in developing countries should consider sedating patients during HSG and documenting observations and patients' feedback to help assess safety and effectiveness in local settings.

## Figures and Tables

**Table 1:** Characteristics of patients undergoing ketamine sedation for HSG.

Characteristics	Frequency, *N* = 27	Percentage (%)
*Age* (*years*)		
Median (IQR)	34 (32–37)	—
25–34	14	51.9
≥35	13	48.1

*Indication*		
Primary infertility	7	25.9
Secondary infertility	20	74.1

*Dosage of Ketamine*		
Median (IQR)	20 (20–30)	—
20 mg/mL	18	66.7
30 mg/mL	4	14.8
40 mg/mL	5	18.5

*Pulse rate before sedation*		
Median (IQR)	65 (64–67)	—
<60 bpm	1	3.7
60–100 bpm	26	96.3
>100 bpm	0	0.0

*Pulse rate when unresponsive*		
Median (IQR)	98 (95–100)	—
<60 bpm	0	0.0
60–100 bpm	23	85.2
>100 bpm	4	14.8

*Pain associated with first HSG*		
No	0	0.0
Yes	27	100.0

*Pain score associated with first HSG*		
9	8	29.6
10	19	70.4

*Pain associated with follow-up HSG under ketamine*		
No	27	100.0
Yes	0	0.0

*Complication associated with ketamine use*		
None	25	92.6
Vomiting	2	7.4

**Table 2:** Distribution of HSG findings by the age group of infertile women under ketamine sedation.

HSG findings	Age group		Fisher's exact test
	25–34 years	≥35 years	*p* value
*Tubal findings*			0.124
Bilateral tubal blockage	5 (41.7)	7 (58.3)	
Bilateral tubal patency	8 (80.0)	2 (20.0)	
RTP, LTB	1 (33.3)	2 (66.7)	
LTP, RTB	0 (0.0)	1 (100.0)	
BH, LLT	0 (0.0)	1 (100.0)	

*Uterine findings*			0.041^*∗*^
Fibroid	0 (0.0)	3 (100.0)	
Normal	14 (60.9)	9 (39.1)	
Irregular/postsurgery	0 (0.0)	1 (100.0)	

^*∗*^Significant level at *p* ≤ 0.05. RTP: right tubal patency; LTP: left tubal patency; LTB: left tubal blockage; RTB: right tubal blockage; BH: bilateral hydrosalpinx; LLT: leakage left tube.

**Table 3:** True positive HSG diagnosis (bilateral tubal blockage).

Bilateral blockage	Initial HSG (A)	Follow-up HSG (B)
Positive	27 (100.0)	12 (44.4%)^#^
Negative	0 (0.0)	15 (55.6%)
Total	27 (100.0)	27 (100.0)

^#^Proportion of true positive bilateral tubal blockage. A: HSG procedure without ketamine sedation. B: repeated HSG procedure under ketamine sedation.

## Data Availability

The data used to support the findings of this study are included within the article.
